# A novel microcurrent dressing for wound healing in a rat skin defect model

**DOI:** 10.1186/s40779-019-0213-x

**Published:** 2019-07-23

**Authors:** Chao Yu, Zhi-Xiu Xu, Yan-Hui Hao, Ya-Bing Gao, Bin-Wei Yao, Jing Zhang, Bing Wang, Zong-Qian Hu, Rui-Yun Peng

**Affiliations:** 1 Department of Experimental Pathology, Beijing Institute of Radiation Medicine, Beijing, 100850 China; 2 Department of Internal Neurology, The First Affiliated Hospital/Xinxiang Medical University, Xinxiang, 453100 Henan China; 3 Department of Biotechnology, Beijing Institute of Radiation Medicine, Beijing, 100850 China

**Keywords:** Microcurrent dressing, Electric stimulation, Skin, Wound healing

## Abstract

**Background:**

The exogenous application of low-intensity electric stimulation (ES) may mimic a natural endogenous bioelectric current and accelerate the repair process of skin wounds. This study designed a novel microcurrent dressing (MCD) and evaluated its potential effects on wound healing in a rat skin defect model.

**Methods:**

First, wireless ES was integrated into a medical cotton cushion to fabricate the MCD, and its electrical property was examined by using a universal power meter. Then, animal experiments were conducted to evaluate the MCD’s effect. Forty-five rats were randomized into control (Con) group, Vaseline gauze (VG) group and MCD group. A full-thickness round skin incision 1.5 cm in diameter was made on the back of each animal. Apart from routine disinfection, the Con rats were untreated, whereas the other two groups were treated with VG or MCD. On days 3, 7 and 14 post injury, the wound areas were observed and measured using image analysis software following photography, and the skin samples were harvested from wound tissue. Then, histopathological morphology was observed routinely by hematoxylin and eosin (HE) staining; tumor necrosis factor α (TNF-α) and interleukin (IL)-1β expression were detected by Western blotting. Vascular endothelial growth factor (VEGF) and epidermal growth factor (EGF) expression were detected with immunohistochemistry.

**Results:**

The MCD generated a sf electric potential greater than 0.95 V. Animal experiments showed that the wound-healing rate in the MCD group was significantly increased compared with the Con and VG groups (*P* < 0.05 or *P* < 0.01). Histopathological observation revealed an alleviated inflammatory response, induced vascular proliferation and accelerated epithelization in the MCD group. Moreover, samples from the MCD group expressed reduced TNF-α and IL-1β levels and increased VEGF and EGF levels compared with those of the other two groups (*P* < 0.05 or *P* < 0.01). However, no significant difference was noted between the Con and VG groups at each time point.

**Conclusions:**

The MCD generates a stable and lasting ES and significantly promotes wound healing by reducing inflammation duration and increasing growth factors expression. Thus, MCD may act as a promising biomaterial device for skin wound healing.

## Background

Wound healing is a complex and dynamic biological event, which includes an inflammation response, tissue formation and remolding. Despite the increasing understanding of the biology of healing, the need for the treatment of skin wounds remains unmet. As a temporary treatment for damaged skin, wound dressings play an important role and are broadly applied to skin wounds. Although medical dressings have improved considerably over time, more biological functions and better treatment effects are required. Technological advances may contribute to this requirement and have promoted the emergence of various wound treatments. Of these treatments, microcurrent therapy or electric stimulation (ES) therapy for wound healing is of interest in this study.

Since microcurrents were first observed at amputation sights [1], the natural electric fields and currents occurring at injury sites have drawn attention and are associated with tissue regeneration and wound healing. With increasing research, numerous lines of experimental and clinical evidence have indicated that the repair process of skin wounds can be accelerated by the exogenous application of low-intensity ES at a physiology level [2–4]. Studies have shown that ES facilitates different stages of the wound-healing process. In the inflammation phase, the application of ES alleviates the inflammatory response and reduces the release of interleukin (IL)-1, tumor necrosis factor α (TNF-α), and nitric oxide (NO) [5–7]. During the proliferation period, ES assists in the expansion of granulation tissue, increases the number of fibroblasts and newly formed blood vessels [8, 9], and induces the expression of growth factors, such as vascular endothelial growth factor (VEGF) and epidermal growth factor (EGF) [9, 10]. During the final remodeling stage, ES contributes to faster epithelization and reduced scar formation [11, 12].

Due to the positive effects of ES, a new treatment strategy is adopted for skin wounds that involves the combination of ES and medical dressing. Currently, several electric dressing devices have been used for skin wounds. Wired devices, such as the POSiFECT® dressing [13] and woundEL® device [14], are examples of these devices; both of these devices are powered by extra electric source and wires. Undoubtedly, this type of exogenous electric supply for dressings restricts their free use. Another type of dressing device is a wireless device that is commercially referred to as a Procellera® dressing [15], which is made of silver/zinc arrays and thin polyester cloth. This device works when the cloth is wet, but the generated ES cannot function for a long period of time given the poor moisturizing ability of its thin polyester cloth. This study designed a novel microcurrent dressing (MCD) based on the principle of the oxidation-reduction reaction. Additionally, the effect of this MCD on skin wound healing was evaluated in a rat skin defect model, and the expression of inflammatory factors (TNF-α and IL-1β) and growth factors (VEGF and EGF) in wound tissue was examined to clarify the potential biological mechanisms.

## Methods

### Production process

The MCD mainly consisted of silver nanoparticles, zinc particles (Aladdin Industrial Inc., China), and a medical cotton cushion. Briefly, the production process proceeded as follows: the silver nanoparticles or zinc particles were fully mixed with a biocompatible agglomerant to generate a silver or zinc slurry with specified concentrations, and the slurries were sprayed on one side of the medical cotton cushion at 2 mm thickness using a dot matrix-arrayed method. The other side of the cushion was covered with thin film that provided moisture and ventilation. After desiccation and sterilization, the MCD was stored hermetically. When used, the MCD was moisten with sterile saline solution or water and then fixed on the wound.

### Experimental animals

Forty-five healthy, male Wistar rats (200~220 g) provided by Laboratory Animal Center of the Academy of Military Medical Sciences were randomized into three groups (15 each): the control (Con) group, the Vaseline gauze (VG) group and the MCD group. All animals were housed one per cage at ambient temperature of 25 °C with a 12-h light/dark cycle. Rats had free access to food and water and were fasted 12 h before the experimental procedure. All animal protocols were approved by the ethics committee of the Academy of Military Medical Sciences.

### Full-layer wound and treatment protocol

All surgical procedures were performed by the same investigator. The hairs on the middle of the back of each rat were shaved, and the area was cleaned with medicinal alcohol. After anesthetization with 1% pentobarbital sodium, a full-thickness round wound with a diameter of 1.5 cm was made on the animal’s back. The coloboma was deepened through the skin and panniculus carnosus to superficial muscle fascia. Normal saline was used to flush the wound. Following disinfection with povidone-iodine, the wounds of the VG group were covered with a piece of VG, whereas the wounds of the MCD group were covered with the MCD, which was moistened with a moderate amount of normal saline beforehand. Wounds in the Con group were left untreated after disinfection with povidone-iodine. The dressings of VG and MCD groups were changed every 3 days. On 3, 7 and 14 days after injury, five rats in each group were sacrificed under anesthesia. Wound areas were measured, and the skin wound healing rate (%) was calculated as follows ([initial wound area-real wound area]/initial area) × 100. Then, the total area of the wound was harvested for laboratory and histological studies. Half of the sample was fixed in 10% formalin buffer for 48 h at room temperature, and the remaining half was stored in a − 80 °C freezer for the following tests.

### Histopathological observation

After routine processing for light microscopy (fixating, dehydrating, embedding, cutting), the tissue specimens (5 each) were mounted on glass slides. Hematoxylin and eosin (HE) staining was used to show inflammatory cells, newly formed vessels, fibroblasts and epithelialization. Images of the sections were captured and digitized using a Leica DM 6000B microscope (Leica, Germany).

### Western blotting

TNF-α and IL-1β expression in wound tissue was detected by Western blotting (5 each and 3 times repeated). TNF-α and IL-1β are mainly expressed in inflammatory cells, and their expression is highest in the early stage of healing, followed by that in the middle stage. Thus, wound tissue samples on days 3 and 7 were chosen for assessment. Skin wound tissue was homogenized in RIPA lysis buffer (Applygen Technology, China) mixed with protease inhibitors, and the protein concentration was determined using the bicinchoninic acid method. Protein extracts were diluted in loading buffer (Solarbio Science&Technology, China) and denatured for 10 min at 95 °C. Equal amounts of protein from each group were separated by 10% sodium-dodecyl sulfate-polyacrylamide gel electrophoresis and blotted to polyvinylidene fluoride microporous membranes (Millipore Corp., MA). After blocking with 5% fat-free milk, the membranes were incubated with primary antibodies against TNF-α and IL-1β (all 1:1000, Santa Cruz Biotechnology, CA) or the internal reference GAPDH (1:5000, Bioworld Technology, USA) overnight at 4 °C. The membranes were then incubated with secondary antibodies, and the immunoreactive proteins were detected using enhanced chemiluminescence reagent (Thermo Fisher Scientific, USA). The generated signals were analyzed with the Image J software (NIH, USA).

### Immunohistochemistry

Immunohistochemistry was performed in wound tissue sections and used to detect VEGF and EGF expression (5 each). VEGF is mainly expressed in endothelial cells, and its levels gradually increase from the early stage to the middle stage of skin wound healing. EGF is mostly expressed in epidermal cells, gradually increasing from the middle stage to the last stage of healing. Thus, the expression of VEGF on days 3 and 7 and EGF on days 7 and 14 after injury were detected. Immunohistochemical reactions were performed using primary antibodies against VEGF and EGF (all 1:200; Bioworld Technology, USA) with overnight incubation at 4 °C. Then, the sections were incubated with secondary antibody (ZSGB-BIO, China) for thirty minutes. Following adequate diaminobenzidine staining and counterstaining with hematoxylin, positive expression could be visualized and recorded by using a microscope. Quantitative analysis of integral optical density (IOD) was performed using Image-Pro Plus software (Media Cybernetics, USA).

### Statistical analysis

All data were presented as the mean ± standard deviation (SD). Statistical analysis was performed using one-way analysis of variance (ANOVA) with Dunnett’s post-hoc test by using SPSS Statistics 19.0 software (SPSS Inc., USA). *P* < 0.05 was considered statistically significant.

## Results

### Microcurrent dressing

**Fig. 1:**
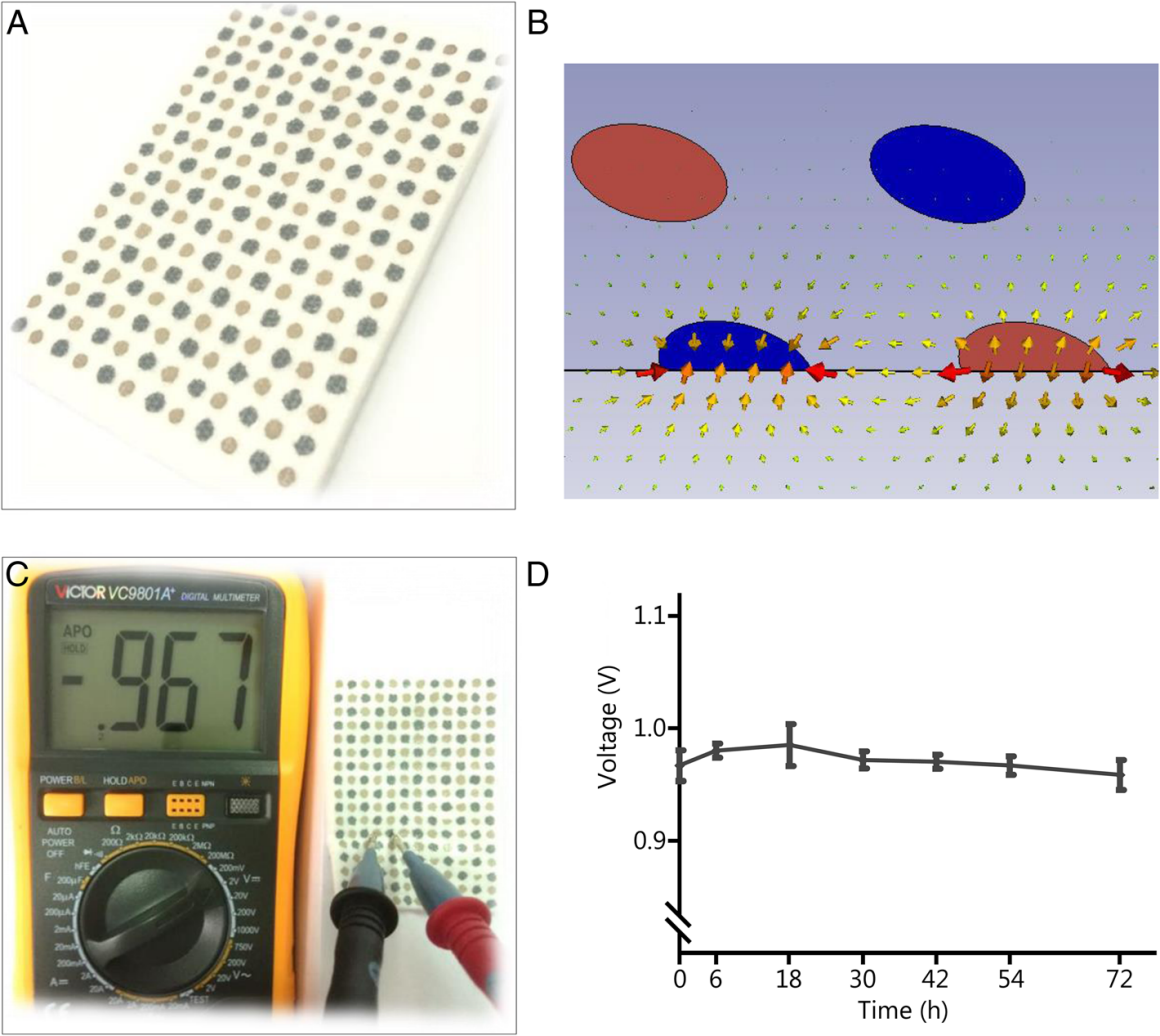
Photographs of the microcurrent dressing (MCD) and the generated electric stimulation. **a.** A real image of the MCD. The different color dots represent different reservoirs. The dark color represents zinc particles acting as reducing agents, and the light color represents silver nanoparticles acting as oxidizing agents. **b.** The distribution of electric fields generated by the MCD. In the presence of moisture, redox reactions occur, and an electric field is generated. **c.** An electric potential between adjacent different electrodes was recorded using a universal power meter. **d.** Changes in electric potentials between adjacent dissimilar electrodes

The MCD was presented as Fig. 1A. When used, the MCD was wet with normal saline, and the moist wound environment promoted the oxidation-reduction reaction between adjacent silver and zinc electrodes (2 mm in distance) (Fig. 1B), which generated an electric potential greater than 0.95 V (Fig. 1C). Accordingly, a field of multiple currents formed across the surface of the dressing substrate. Surprisingly, the measurement results showed that the MCD could exert stable and lasting (3 days or more) ES without supplying electricity (Fig. 1D).

### MCD improved the wound-healing rate

**Fig. 2:**
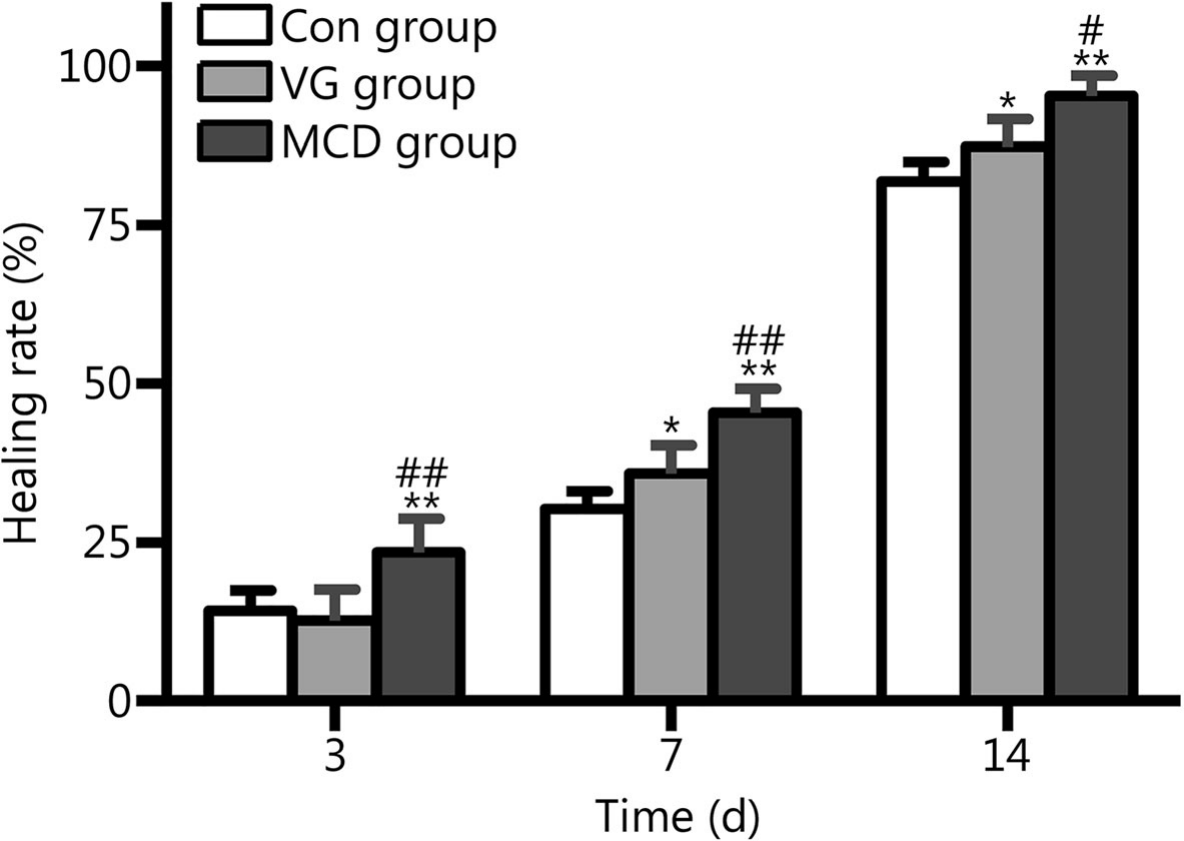
Wound healing rate (percentage) on 3, 7 and 14 days post injury. The MCD group exhibited a significantly increased healing rate compared to other two groups on day 3. On days 7 and 14, the MCD group exhibited superior healing rates when compared with the VG group, and the healing rates in both of groups are higher than those of the Con group. ^*^P < 0.05 and ^**^P < 0.01 compared with the Con group; ^#^*P* < 0.05 and ^# #^*P* < 0.01 compared with the VG group

On day 3 post wounding, the healing rates of the wounds in the Con group, VG group and MCD group were 14.16% ± 3.18, 12.61% ± 4.96 and 23.40% ± 5.26%, respectively, and the rate in the MCD group was significantly higher than those of the other groups (*P* < 0.01). No difference was noted between the Con and VG groups (*P* > 0.05). On day 7, the healing rate in the MCD group was 45.32% ± 3.80%, which exceeded the rate obtained for the Con group, 30.27% ± 2.72% (*P* < 0.01), and the rate obtained for the VG group, 35.88% ± 4.46% (*P* < 0.01). On day 14, the Con group healing rate was 81.88% ± 3.07%, which was lower than that of the VG group, 87.31% ± 4.29% (*P* < 0.05), and the MCD group, 95.32% ± 3.20% (*P* < 0.01); the VG group healing rate was also lower than that of the MCD group (*P* < 0.05, Fig. 2).

### MCD alleviated the inflammatory response, induced vascular proliferation and accelerated epithelization

**Fig. 3:**
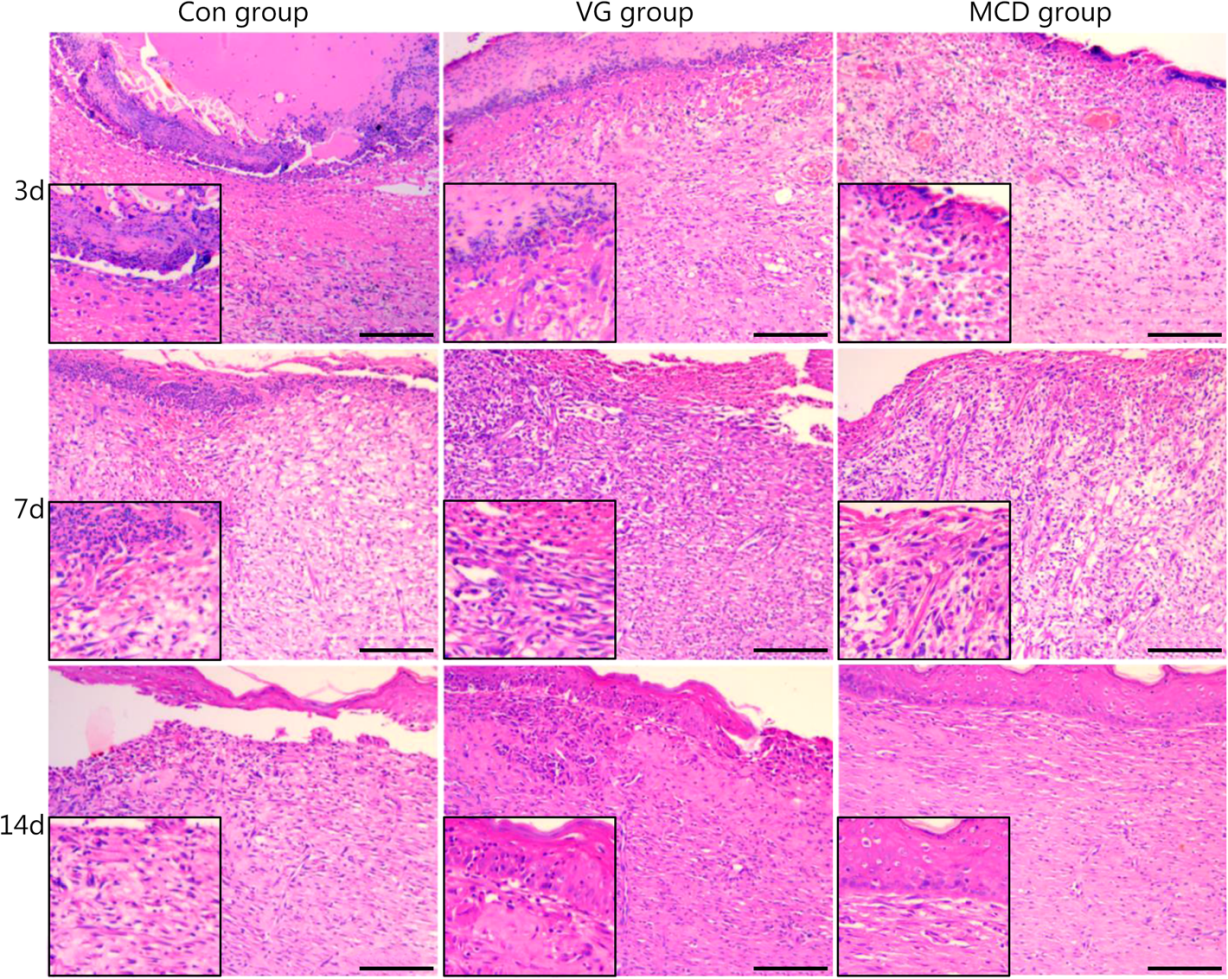
Light microscopy images of full-layer wounds (HE, scale bar = 100 μm). HE histological observation of wounds without dressing in the control group (Con) and wounds covered with Vaseline gauze (VG) and microcurrent dressing (MCD). The left bottom images present magnified views

Histological observations of wounds in different groups are presented in Fig. 3. On day 3, the wound site was characterized by an inflammation response and filled with inflammatory cells. In the Con group, the broad accumulation of inflammatory cells and thick escharosis outside of the wound was distinct due to the abundant exudation of tissue fluid and necrosis of superficial cells. A moderate inflammation response was observed in the VG group. The response extent was significantly alleviated in the MCD group. On day 7, granulation tissue was evident in the dermis, and blood capillary formation was the primary biological event. An increased extent of proliferated vasculum was observed in the MCD group compared with the Con and VG groups. As the wound continued to heal on day 14, the number of fibroblasts gradually decreased as did fibrous composition, and the capillaries closed and gradually disappeared. In addition, epidermal cells migrated to cover the entire wound, and the epithelization process gradually ended. The epidermis and dermis in the Con group were loosely packed. An immature epidermis was observed in the VG group, while the MCD group was well re-epithelialized and accompanied by a layer of keratin.

### MCD reduced the expression of inflammatory cytokines

**Fig. 4:**
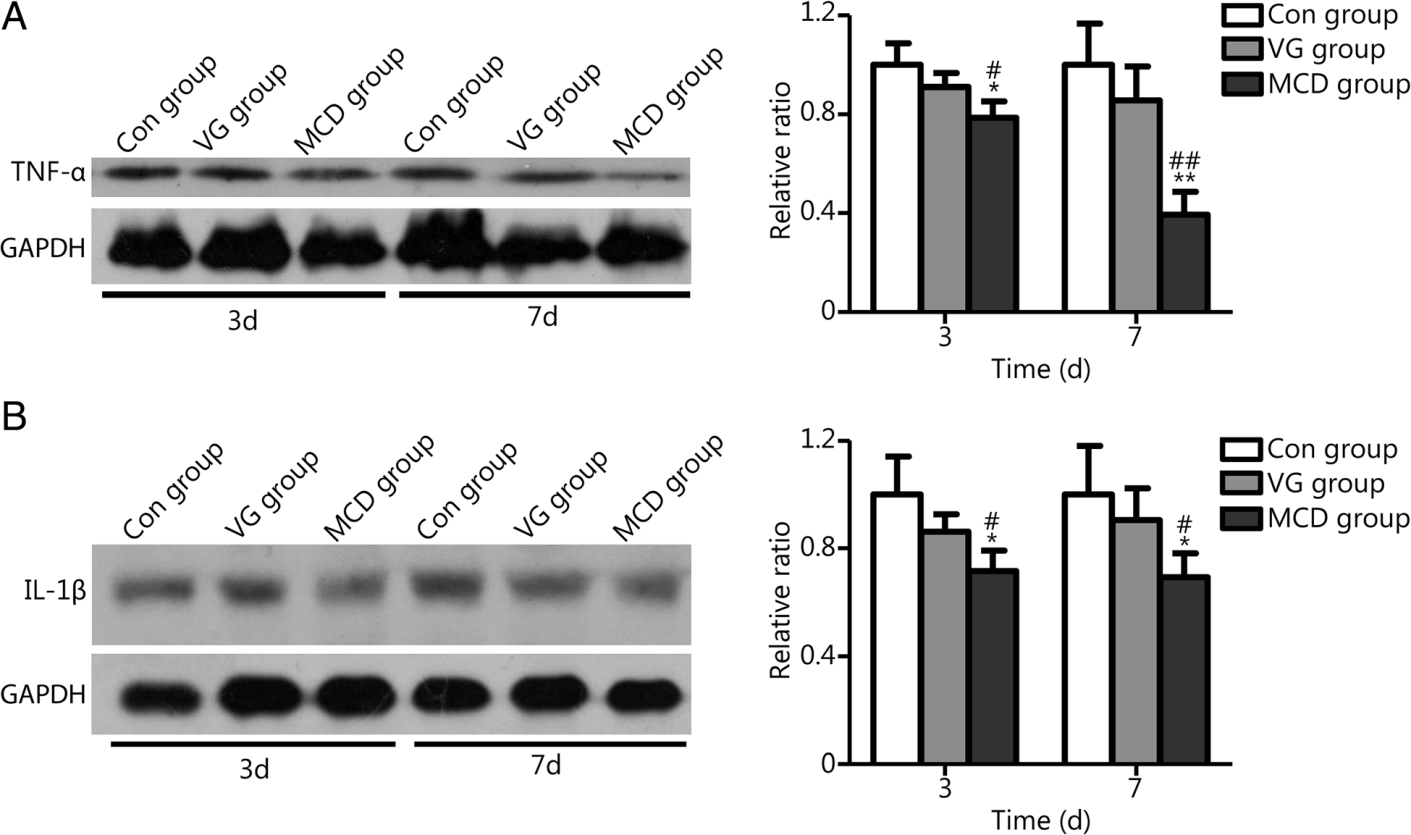
TNF-α and IL-1β expression in Con, VG and MCD groups. Chemiluminescence was quantified with Image J software to calculate the ratio to GAPDH. The MCD group exhibited lower TNF-α and IL-1β levels compared to the other two groups on days 3 and 7. No significant difference was observed between Con and VG groups. ^*^P < 0.05 and ^**^P < 0.01 compared with Con group; ^#^*P* < 0.05 and ^# #^*P* < 0.01 compared with VG group

To analyze the difference in inflammatory cytokine expression in untreated and treated wounds, the relative levels of TNF-α and IL-1β on days 3 and 7 post wounding were detected by Western blotting. Chemiluminescence was quantified with Image J software to calculate the ratio to internal reference GAPDH. The results are presented in Fig. 4. Compared with the respective Con group, significant decreases in TNF-α (Fig. 4A) and IL-1β (Fig. 4B) expression were noted in the MCD group on days 3 and 7 (*P* < 0.05 or *P* < 0.01). No significant difference was noted between the Con and VG groups (*P* > 0.05).

### MCD increased the expression of growth factors

Growth factors play an important role in regulating wound healing, and VEGF and EGF are major players. Thus, their expression levels were analyzed to evaluate the effect of MCD dressing.

**Fig. 5:**
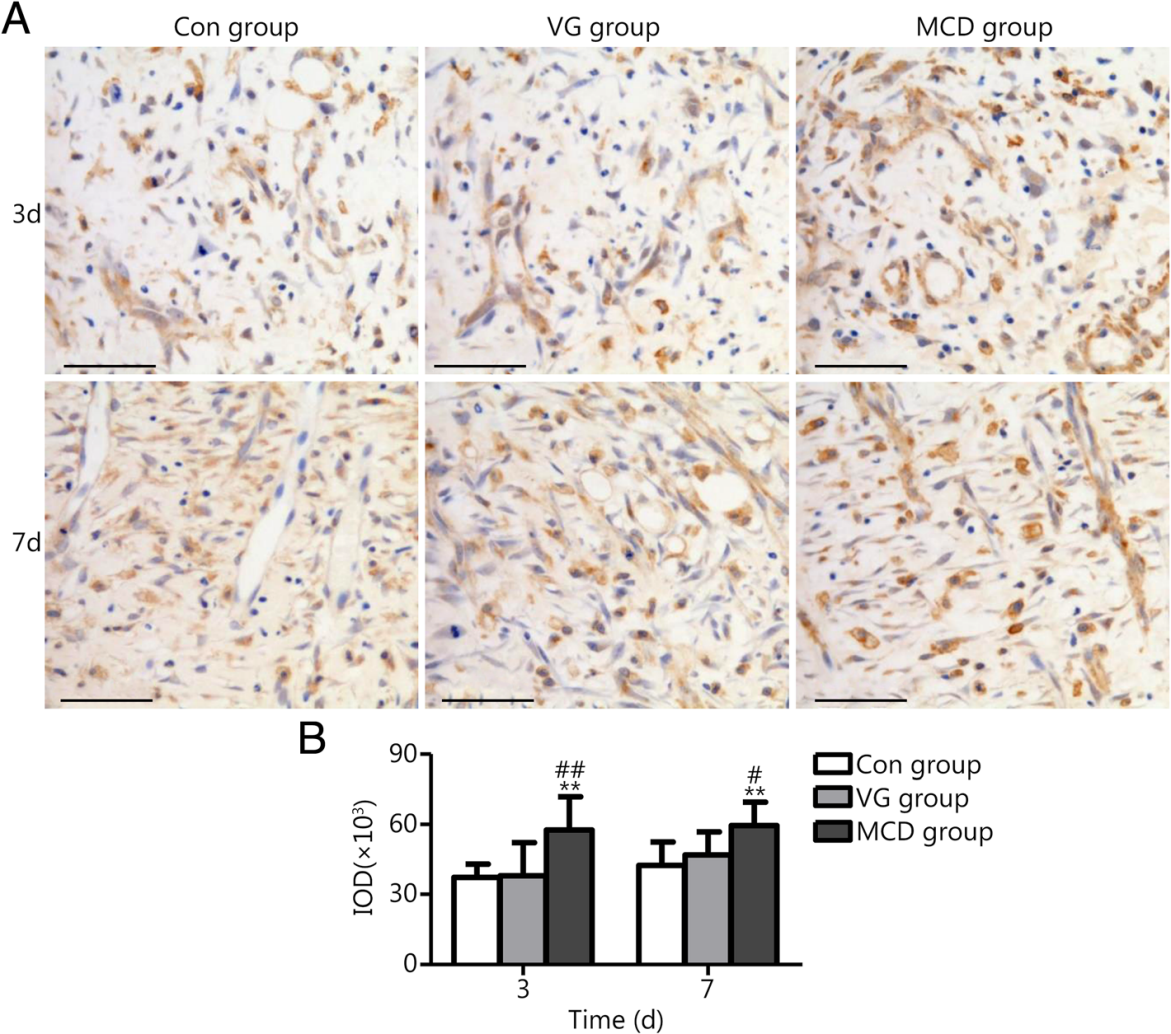
VEGF expression in Con, VG and MCD groups. **a**. Light microscopy images as assessed by immunohistochemistry (scale bar = 50 μm). **b**. Quantitative analysis results of IOD as assessed by Image-Pro Plus software. The MCD group exhibited higher expression levels compared with the other groups on days 3 and 7. No significant difference was observed between the Con and VG groups. ^**^P < 0.01 compared with the Con group; ^#^*P* < 0.05 and ^# #^*P* < 0.01 compared with the VG group

**Fig. 6:**
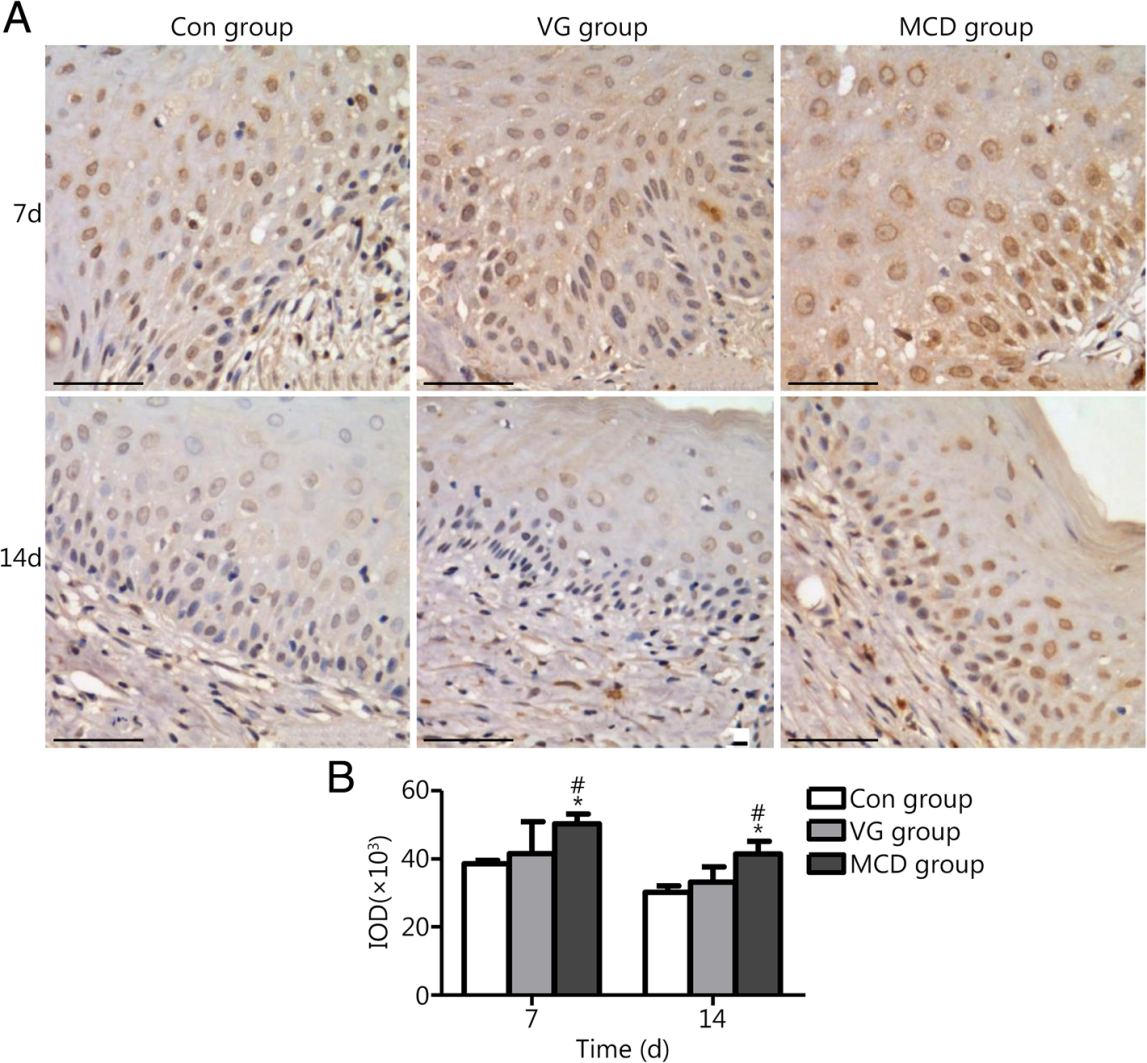
EGF expression in the Con, VG and MCD groups. **a**. Light microscopy images as assessed by immunohistochemistry (scale bar = 50 μm). **b**. Quantitative analysis results of IOD as assessed by Image-Pro Plus software. The MCD group exhibited higher expression compared with other groups on days 7 and 14. No significant difference was observed between the Con and VG groups. ^*^P < 0.05 compared with the Con group; ^#^*P* < 0.05 compared with the VG group

VEGF and EGF expression in wound sites was assessed by immunohistochemistry. The histological observations are presented in Figs. 5 and 6. Abundant VEGF expression was present in the plasma of endothelial cells (Fig. 5A). Combining the photographs and statistical results, the expression levels of VEGF in the MCD group were significantly higher than those of the other two groups on days 3 and 7 (*P* < 0.05 or *P* < 0.01). Differences were rarely noted between the Con and VG groups (*P* > 0.05). EGF expression is shown in Fig. 6. The MCD group showed higher EGF expression in epidermal cells when compared with the other groups on days 7 and 14 (*P* < 0.05). No significant difference was observed between the Con and VG groups.

## Discussion

This study designed a novel MCD made of zinc particles, silver nanoparticles and medical cotton cushion, all of which are widely used in the clinic. As an indispensable trace element in the human body, zinc plays a significant role in health and disease. Various zinc-containing products are available for topical application in wound management, such as zinc oxide compression dressings and zinc-hyaluronic gel [16, 17]. In addition, silver and its compounds have been employed for centuries due to their medicinal performance, especially the effects on wound infection control. At present, various silver-based dressings or other wound care productions have emerged as an effective treatment option for infections in burns, open wounds and chronic ulcers [17, 18]. In this research, zinc particles and silver nanoparticles mixed with a biocompatible agglomerant were sprayed on the surface of medical cotton cushion in a dot matrix form to generate low-level ES at the device-wound contact surface in the presence of moisture without the need for any external power supply.

This study mainly focused on the evaluation of the biological effects of MCD. Since Becker reported the “currents of injury” [1], ES has been investigated and applied in human healthcare and is currently employed in pain control, cancer treatment and wound healing [19–21]. The beneficial effects of ES in human and animal wound healing models and on cells involved in wound healing have been shown in many studies. In 2002, the Centers for Medicare and Medicaid Services approved reimbursement for use of ES in a clinical setting for certain chronic wounds [22].

In this study, analysis of the wound-healing rate revealed increased levels in the MCD group, indicating the effectiveness of MCD. More healing parameters were analyzed by histological changes. Several studies have detected the effects of ES on histological parameters at the wounded tissue. Komegae et al. [23] reported that ES led to a reduction in inflammation responses. Passarini et al. [24], Castro et al. [25], and Fernanda et al. [26] described an increase in the number of newly formed blood vessels and in the progress of epithelization in wounds experimentally induced in Wistar rats treated with microcurrent. Similar results were noted in this study. Reduced inflammatory cell accumulation and escharosis on day 3, increased angiogenesis on day 7, and mature epidermis on day 14 were found in the MCD group compared with the Con and VG groups. Thus, we believed that MCD application was effective in terms of gross observation and histological performance.

Further, reduced inflammatory cytokine expression was observed in wounds treated with MCD, which was consistent with the histological results and the literature [5, 27]. Although an initial inflammatory response positively promotes the body to clean infection or pathogens from our tissue, hyperactive and long-term inflammatory states are closely associated with chronic non-healing wounds. Moreover, the inflammatory phase of healing is absent in scarless fetal wound healing [28]. TNF-α and IL-1β are inflammatory cytokines that play important roles in the early phase of inflammation in the wound-healing process. In MCD rats, the reduction in TNF-α and IL-1β expression after treatment on days 3 and 7 suggested decreased inflammation, which facilitated healing.

In addition, the regulation of growth factors (e.g., VEGF and EGF) alters wound healing. Numerous cell types, including inflammatory cells, fibroblasts, and endothelial cells, release these factors. VEGF regulates the multiple biological functions of endothelial cells that give rise to the production of angiogenesis, and EGF accelerates the growth of keratinocytes to increase epidermal regeneration. In this study, increased VEGF levels on 3 and 7 days and increased EGF levels on 7 and 14 days were noted in MCD rats. These results indicated the promoting effect of MCD on growth factor expression.

The exact mechanisms by which ES influences the expression of inflammatory cytokines and growth factors are not well characterized. Regulation of the inflammation response is closely related to nuclear factor-κB (NF-κB) activity [29], and studies found that the suppression of NF-κB activity by ES is one of the potential pathways to reduce inflammation [27]. Additionally, the activation of bone morphogenetic protein (BMP)/Smad, phosphatidylinositol 3-kinase/protein kinase B (PI3K/PKB), epidermal growth factor receptor (EGFR) and mitogen-activated protein kinase (MAPK) signaling pathways induced by ES was associated with cell migration and wound healing [30–33]. However, whether these signals are involved in skin wound healing induced by MCD remain unknown, and further studies are required.

## Conclusions

The results obtained in this work showed that the MCD had a significant impact on the healing process and could accelerate wounding healing. Despite the good performance, design improvements and further evaluation of MCD are needed. The evaluation of wound types (e.g., burn, radiation injury or chronic ulcers) and clinical studies are needed to further investigate the potential application of MCD as a promising biomaterial device for skin wounds.

## Data Availability

All materials are commercially available, and data are presented in this article are available per the open access policy.

## Abbreviations

ANOVA: Analysis of variance
BMP: Bone morphogenetic protein
Con: Control
EGF: Epidermal growth factor
EGFR: Epidermal growth factor receptor
ES: Electric stimulation
HE: Hematoxylin and eosin
IL-1: Interleukin-1
IOD: Integral optical density
MAPK: Mitogen-activated protein kinase
MCD: Microcurrent dressing
NF-κB: Nuclear factor-κB
NO: Nitric oxide
PI3K/PKB: Phosphatidylinositol 3-kinase/protein kinase B
SD: Standard deviation
TNF-α: Tumor necrosis factor α
VEGF: Vascular endothelial growth factor
VG: Vaseline gauze
